# Chitosan capped-NLCs enhanced codelivery of gefitinib and simvastatin into MDR HCC: impact of compositions on cell death, JNK3, and Telomerase

**DOI:** 10.32604/or.2024.053337

**Published:** 2025-01-16

**Authors:** GAMALELDIN I. HARISA, RIYAD F. ALZHRANI, ABDULRAHMAN A. ALLUHAIDAN, SULTAN M. ALAMRI, AHMED H. BAKHEIT, HANADI H. ASIRI, SABRY M. ATTIA

**Affiliations:** 1 Department of Pharmaceutics, College of Pharmacy, King Saud University, Riyadh, 11451, Saudi Arabia; 2 Department of Pharmaceutical Chemistry, College of Pharmacy, King Saud University, Riyadh, 11451, Saudi Arabia; 3 Department of Pharmacology and Toxicology, College of Pharmacy, King Saud University, Riyadh, 11451, Saudi Arabia

**Keywords:** Codelivery, Chimeric therapy, Hepatocellular carcinoma, Multi-drug resistance, Telomerase

## Abstract

**Background:**

Hepatocellular carcinoma (HCC) is a health problem due to multi-drug resistance (MDR). Codelivery of multiple oncotherapy in one cargo as chimeric cancer therapy (CCT) is suggested as a solution for MDR. This study aims to engineer chitosan-coated nanostructure lipid carriers (NLCs) loaded with gefitinib (GF) and simvastatin (SV) as CCT for HCC.

**Methods:**

Both GF and SV-loaded nanostructure lipids carriers (GFSVNLC) and chitosan-capped GF and SV-loaded nanostructure lipids carriers (CGFSVNLC) formulations were assembled by top-down techniques. Moreover, particle size (PS), zeta potential (ZP), and polydispersity index (PDI) were measured by Zetasizer. The biosafety of GFSVNLC preparations was investigated by using erythrocytes as a biological model. The cytotoxic, and apoptotic effects of the prepared GFSVNLCs were investigated using HepG2 cell lines as a substitute model for HCC. The effect of GF, SV, and NLC composition on JNK3, HDAC6, and telomerase was studied using molecular docking simulation (MDS).

**Results:**

The present results revealed that the obtained GFSVNLC and CGFSVNLC have nanosized and consistent, CS coating shifts anionic ZP of GFSVNLC into CGFSVNLC with cationic ZP. Moreover, both formulations are biocompatible as indicated by their gentle effect on erythrocyte hemolysis. The treatment of HepG2 cells with GFSVNLC, and CGFSVNLC induced marked cell death compared to other groups with a decrease of IC50. Equally, the percentage of the apoptotic HepG2 cells was increased upon treatment of the cells with GFSV, GFSVNLC, and CGFSVNLC compared to the control group. Additionally, GF, SV, stearic acid (SA), and oleic acid (OA) modulate the activity of JNK3, HDAC6, and telomerase.

**Conclusions:**

This study suggests CGFSVNLC achieves codelivery, selective targeting, and enhancing the synergistic effect of GF and SV for inducing HepG2 cell death. Mechanistically, CGFSVNLC inhibits key cascades implicated in MDR and HepG2 cell survival. CGFSVNLC is promising for overcoming drug resistance mechanisms and improving therapeutic outcomes against HepG2 cells.

## Introduction

Multi-drug resistance (MDR) is a major problem in the management of breast, ovarian, lung, gastrointestinal, hematological cancer, and hepatocellular carcinoma (HCC) [1]. MDR is a phenomenon in which tumor cells progress their ability to resist the effect of cytotoxic drugs chemotherapeutic drugs [2]. MDR of tumor cells to chemotherapy is achieved by ample cellular mechanisms [3,4]. These types of machinery include modification of anticancer targets, efflux pumps, detoxification, and programmed cell death machinery [4–6]. MDR can be mediated by several genetic and epigenetic mechanisms. The common genetic machinery of MDR includes overexpression of drug efflux pumps, mutations in drug targets, and alterations in DNA repair mechanisms [2]. However, the epigenetic machinery of MDR includes the alteration of histone modifications, DNA methylation patterns, and gene editing [2]. Moreover, alteration of drug metabolism, apoptosis, cellular senescence, cancer stem cells, and autophagy are involved in MDR [2].

HCC is the fourth leading cause of cancer-related human expiry worldwide [7]. HCC is the most common type of primary hepatic cancer, HCC is classified as solid and originates from hepatocytes that account for approximately 90% of HCC cases. HCC cells can proliferate and metastasize to other bod [7]. Liver diseases such as cirrhosis, viral hepatitis infections, alcohol addiction, and non-alcoholic fatty liver diseases could mediate the malignant transformation of hepatocytes into HCC [7]. HCC therapeutic problem resulted from MDR to classical and targeted chemotherapy [4]. Frequently, MDR induces failure in the therapeutic outcome of anticancer agents, thus, HCC patients are at high risk of death [1,8]. Chimeric cancer therapy (CCT), immunotherapy, phototherapy, and nanomedicine were exploited as hopeful strategies for the prevention of MDR by malignant cells [2,9]. CCT and nanoscale drug delivery cargoes (NDCs) are proposed as innovative plans to control HCC and prolong HCC patients [10]. For instance, effective cancer therapy should target several cellular processes such as metabolic, genetic, and signaling pathways [11]. Herein, the use of CCT is a plan for the management of cancers and combat MDR [12].

CCT could target multiple processes involved in cancer cell growth signal cascade to give a satisfying therapeutic impact due to overwhelming MDR [3,5]. Additionally, CCT harnesses codelivery techniques in which 2 or more drugs are loaded in 1 drug delivery cargo. Thus, CCT could evade the MDR by tumor cells [1,5]. Likewise, CCT enhances drug development, improves pharmacokinetic profile, and reduces the risk of drug-drug interactions [3]. In this regard, CCT is designed to resolve HCC therapeutic problems, however, it offers selective delivery as well as attenuates cancer-induced MDR [8,13].

Commonly, HCC is treated with tyrosine kinase inhibitors (TKIs) to block the over-functional tyrosine kinase that mediates the hysterical proliferation of cancer cells [14,15]. TKIs stop the phosphorylation of growth factor receptors by chelation of the magnesium ions (Mg) and ATP as an essential cofactor for tyrosine kinases, telomerase, and c-Jun N-terminal kinase-3 (JNK3) and other MAPK [16,17]. Interfering with kinases control cell proliferation, cell senescence, differentiation, survival, and apoptosis [18]. Likewise, alteration of histone deacetylase (HDACs) enzymes and telomerase activities are associated with cancer progression [19–21]. Thus, HDAC inhibition is a hopeful plan for cancer control, moreover, the combination of HDAC inhibitors and conventional chemotherapy could induce synergistic effects with improved therapeutic impact [22]. As well, bioinformatics analysis could be a novel gene signature as a model for possible therapeutic modalities of HCC to sustain the survival of hepatic cancer patients [23].

Gefitinib (GF) is a member of the TKIs family that is useful in the treatment of HCC, otherwise, GF has shown numerous systemic toxicities due to high dosing and MDR [14]. Accordingly, codelivery and repurposing of non-oncology small-molecule drugs have been reported as an approach to overcome MDR [24]. It has been demonstrated that the double therapy TKIs such as sorafenib and phototherapy amplified the chemosensitivity of human hepatic cancer cells to anticancer medicines [9]. In this regard, statins such as simvastatin (SV) are repurposed as anticancer agents due to inhibition of cholesterol, and mevalonate production pathways as dynamic molecules for cellular proliferation. Moreover, statins affect the growth at the epigenetic level [25]. In this context, ample studies repurposed SV alone or in combination to improve patient prognosis of HCC and other human cancers [12,26]. Despite this, SV was repurposed as chemo-preventive, monotherapy, or in combination with TKIs as HCC therapy [5,12,24,25,27], Further studies are required to confirm this theme.

Unfortunately, both GF and SV are suffering from low solubility, high dosing, MDR, and low bioavailability. Thus, NDCs are suggested to simplify GF and SV delivery into cancer cells [5,28]. The NDCs such as lipid nanoparticles and others augment the bioavailability of oncotherapies with control drug release as well as drug targeting ability, this alleviates systemic toxicity. Thus fabrication of NDCs is potential for upcoming developments in malignancy control [29]. The assembly of NDCs loaded by several oncotherapies improves the clinical outcome, thus, CCT induces synergistic drug actions and inhibits MDR [1]. From the drug delivery point of view, nanostructured lipid carriers (NLCs) have high drug entrapment, control drug release, are stable, and can be produced at a large scale [30,31]. Certainly, the drug impeded in NLC core is highly protected with improved bioavailability, codelivery, and tumor targeting [31]. Nanotechnology studies showed that, the concurrent delivery of GF and SV into NLCs payload induced drug co-localization within the tumor microenvironment by passive and active machinery [28,32]. The main ingredients of NLC formulations are fatty acids (FAs) such as stearic acid (SA), oleic acid (OA), palmitic acid, or other FAs that are highly uptake by cancer cells. Consequently, NLCs enriched FAs could be harnessed as clever anticancer drug delivery systems [33]. Importantly, zeta potential (ZP) influences the import of NLCs by the cancer cells. Indeed, the outward of NLC cargoes could be engineered to become neutral, cationic, or anionic [34].

Chitosan (CS) is utilized to induce cationic ZP on NLC, besides this, CS elicits cholesterol-lowering, antioxidant, immunomodulatory, and anticancer action [35]. CS is used for the surface engineering of nanoparticles due to biocompatibility, and biodegradability [36]. The surface engineering of NLCs by CS-induced positive ZP enhances cellular uptake and improves the therapeutic effect [36]. Due to mucoadhesive properties, CS-engineering of lipid nanoparticles extends the contact time with the cells [37]. Indeed, CS engineering of NLC-induced cationic corona could improve the therapeutic impact of nanomaterials [38].

Taken together, CCT could induce a positive therapeutic impact of cytotoxic agents for example, several studies documented that, SV increases the sensitivity of cancer cells to TKIs by inhibition of MDR. Thus, the co-delivery drug approach harnesses CCT to target ample cascades that provoke malignant transformation with inhibition of MDR. Accordingly, the current study aims to study the effect of CS-capped NLC-loaded GF and SV (CGFSVNLC) on HCC mortality. CGFSVNLC cargoes were prepared and characterized in terms of particle size (PS), ZP, and polydispersity index (PDI) using Zetasizer. Next, the biocompatibility was investigated using blood samples. Then, the cytotoxicity and apoptotic effect of CGFSVNLC chimeras were investigated using HepG2 cell lines as a surrogate model for HCC. After that, the influence of GF and SV as active pharmaceutic ingredients, besides SA, OA, and CS as NLC components on JNK3, HDAC6, and telomerase was studied using molecular docking simulation (MDS).

## Materials and Methods

### Materials

GF was purchased from Beijing Mesochem Technology Co., Ltd. (Beijing, China). SV was supplied as a gift from Riyadh Pharma Company, Riyadh, Saudi Arabia. SA was obtained from BDH (Poole, UK). OA was acquired from Avonchem (Cheshire, UK). CS low molecular weight (MW, 100–150 kDa; degree of deacetylation, 85%) and Pluronic F-68 (MW 8.40 kDa) were obtained from Sigma Aldrich (St. Louis, MO, USA). Entirely additional substances were presented in analytical marks.

### Manufacture of NLCs

A previously reported ultrasonic melt-emulsification method was utilized to prepare plain NLCs **(**PNLC), GFSV-loaded NLCs (GFSVNLC), CS-coated PNLCs (CPNLC), and CS-engineered GFSVNLC (CGFSVNLC) [39]. The components of all formulations are indicated in Table S1. Firstly, the precise amount of SA as solid lipid, OA as liquid lipid, GF, and SV were weighed and placed in a cylindrical beaker to make the lipid phase. In another beaker, Pluronic F-68 as surfactant and water were placed to prepare the aqueous phase. The Pluronic F-68 was left at 4°C for a complete solution. Next, both lipid and aqueous phases were simultaneously heated up to 80°C. Then, the hot aqueous phase was added to the hot lipid phase and then stirred together to prepare the primary micro-emulsion. Finally, NLC preparations were obtained after sonication of the primary micro-emulsion using a probe sonicator at 80% voltage efficiency for 6 cycles. Each cycle extended for 40 s disrupted with a resting period extended for 5 s. The consistent milky appearance of the preparations was used as an indicator for NLC production. The assembled NLC formulations were kept in a cool place for further use.

### CS capping of GFSVNLC

CS-capped GFSVNLC (CGFSVNLC) were engineered by mixing equal volumes of NLC, and CS solution. The latter was prepared by dissolving 1% CS in 0.5% acetic acid solution pH was adjusted at 5.5–6.0. The CS solution was added dropwise to NLCs under magnetic stirring for 20 min. The resulting formulations were maintained under continuous stirring for 2 h to obtain CNLC [40]. The obtained CNLC preparations were kept in a cool place for further use.

### Characterization of NLCs

PS, PDI, and ZP of prepared PNLC, GFSVNLC, CPNLC, and CGFSVNLC were characterized using a Zetasizer Nano ZS (Malvern Instruments, Malvern, UK). Each formulation was diluted (1:1000) in phosphate-buffered saline and assessed at 25°C. Dynamic Light scattering and Laser Doppler Velocimetry modes were utilized to measure PS and ZP, respectively. Each sample was measured in triplicate [41].

### Biosafety studies

The blood suspension was utilized to investigate biosafety in terms of hemocompatibility of the drug delivery system as described by Harisa et al., with some modifications [30]. The blood sample was diluted with physiological saline solution to obtain 2% blood suspension. The blood suspension was incubated with the solution of free drugs as well as drug-loaded NLCs.

Group 1: The blood suspension was treated with 1% DMSO as a control group. Group 2: The blood suspension was treated with GF dissolved in 1% DMSO. Group 3: The blood suspension was treated with SV dissolved in 1% DMSO. Group 4: The blood suspension was treated with the combination of GF and SV dissolved in 1% DMSO. Group 5: The blood suspension was treated with PNLCs. Group 6: The blood suspension was treated with GFSV-NLCs. Group 7: The blood suspension was treated with CPNLCs. Group 8: The blood suspension was treated with CGFSVNLC.

In the negative control group, with no hemolysis, the blood suspension was incubated with phosphate-buffered saline, however, in positive control, complete hemolysis, blood suspension was incubated with distilled water to induce hypotonic erythrocyte lysis. The incubated blood suspension in all groups was mixed by gentle inversion several times and kept at 37°C for 1, 24, 48, and 72 h. Afterward, the samples were centrifugated at 3000 rpm for 5 min, and absorbance was measured at 570 nm. The hemolysis percentage was calculated using the following equation:

$$\begin{array}{rl} & Hemolysis\;=\\ & \frac{Absorbance\;sample-absorbance\;negative\;control}{\;absorbance\;positive\;control-absorbance\;negative\;control}\\ & *100 \end{array}$$

### Cell culture and HepG2 cell mortality studies

HepG2 cell line as an alternative model for HCC to investigate the cytotoxicity of free GF, SV alone or in combination, besides, GFSV loaded NLCs with or without CS engineering. HepG2 cells were acquired from the American Type Cell Culture (ATCC, Manassas, VA, USA). The cell line was supplemented with Dulbecco’s modified Eagle’s medium, 1%v/v penicillin-streptomycin, and 10%v/v fetal bovine serum. After that, cultured cells were kept in a humidified incubator at 37°C with 5% CO_2_. Briefly, HepG2 cells were seeded in 96-well culture plates at a density of 2 × 10^4^ cells/well using a 96-well for 24 h before the experimentation. The cultivated cells were treated with free drugs and NLC-loaded drug preparations as follows:

Group 1: HepG2 cells were treated with 1% DMSO as a control group. Group 2: HepG2 cells were treated with GF. Group 3: HepG2 cells were treated with SV. Group 4: HepG2 cells were treated with a combination of SV and GF, free GF, and SV, and their combination was dissolved in 1% DMSO. Group 5: HepG2 cells were treated with PNLC formulations. Group 6: HepG2 cells were treated with GFSVNLC. Group 7: HepG2 cells were treated with CPNLCs. Group 8: HepG2 cells were treated with CSVGFNLC preparations. The doses of GF and SV were selected based on the previously published work [5].

All groups were incubated for 24, 48, and 72 h. The percent of cell viability was detected using an MTT assay. Finally, the cells were treated with 50 μg of MTT and kept at 37°C for 4 h in the dark. Then, cells were treated with acidified isopropanol to enhance the solubilization of the formazan product. The absorbance of the formazan product was quantified at 570 nm wavelength using a microplate reader (Bio-Tek, Winooski, VT, USA). A dose-response curve was achieved by plotting drug concentration against cell viability where IC_50_ was calculated.

Cell viability (%) was calculated using the following equation:

$$Viability\;\left(\%\right)=\frac{optical\;density\;of\;treated\;sample}{\;optical\;density\;of\;untreated\;sample}*100$$

### Apoptosis studies

The effect of GF, SV solution alone or combination in 1% DMSO, as well as GFSVNLC and CGFVSNLC on HepG2 programmed cell death, was investigated using an annexin V-fluorescein isothiocyanate (FITC)/propidium iodide apoptosis detection kit (Sigma, Livonia, MI, USA). Firstly, HepG2 cells were seeded into 12-well plates at a density of 1 × 10^6^ cells/well in 1 mL Dulbecco’s modified Eagle’s medium for 12 h. Next, HepG2 cells were incubated with GF, SV, GFSV, GFSVNLCs, and CGFSVNLCs. Next, the cells were incubated for 24 h, then they were washed with phosphate-buffered saline and lysed with trypsin. Then, the cells were harvested by centrifugation at 2000 × *g*. Subsequently, cells were diluted by binding buffer with double distilled water, and 500 μL of suspension cells with 1 × binding buffer were prepared. After that, 500 μL of treated and the non-treated cell suspension was incubated with 5 μL of Annexin V-FITC and 10 μL of propidium iodide at room temperature for 30 min in darkness. Finally, the fluorescence of the cells directly was determined using a flow cytometer (Beckman Coulter, CA, USA).

### Molecular docking simulation

MDS is used as a support approach in predicting of therapeutic effect of the compounds. The present study employed an advanced molecular docking technique, a tool increasingly utilized within the pharmaceutical research community, to estimate the inhibitory potential of synthesized compounds or extracts derived from natural sources. This approach is critical in assessing the efficacy of these compounds before they are applied in therapeutic contexts. The MOE-docking module (2015 version) was utilized to perform docking studies on the 2P33, 6PYE, and 5CQG proteins, formatted in PDB. The chosen protein, JNK3 (PDB: 2P33) [42], HDAC6 enzyme (PDB: 6PYE) [43], and telomerase (PDB: 5CQG) [44] are notable for its elevated presence in cancer cells compared to normal cells. The docking procedure was governed by specific protocols. Initially, we conducted energy minimization for each compound’s GF, SV active metabolites (tenivastatin), SA, OA, and the co-crystalline ligand. This step was followed by assigning charges to the atoms, adjusting potential energy, and setting the protonation state at pH 7.4, using the MOE Protonate 3D. Subsequently, each oriented compound was stored in an MDB format database for further analysis [45].

The molecular mechanics force field MMFF94x was employed to control additional parameters. Construction of each protein co-crystal involved adding hydrogen atoms to particular receptors and defining receptor connections. After stabilizing the potential energy, we used a co-crystal ligand as a reference point for the docking site. The docking simulations generated 30 poses per run, regulated by the London dG scoring function and refined using the Triangle Matcher algorithm. Each simulation’s outcome was analyzed for interaction data, binding patterns, and surface mapping, which were crucial in determining the inhibitory efficiency. Authentic interactions were validated based on hydrogen bond lengths, which should not exceed 3.5 Å.

We set the placement method and scoring function to Triangle Matcher and London dG, respectively, while the refinement method and scoring function were adjusted to Induced Fit and GBVI/WSA dG. These methodological choices were informed by current best practices in molecular docking studies, ensuring accurate and reliable predictions of compound efficacy.

### Statistical analyses

Data analysis was achieved by GraphPad software, version 5 (GraphPad, ISI Software Inc., La Jolla, CA, USA). The results were compared using a one-way analysis of variance. Data were expressed as mean ± SD and *p*-value < 0.05 were used as criteria for significance.

## Results

### Impact of NLCs composition

**Figure 1:**
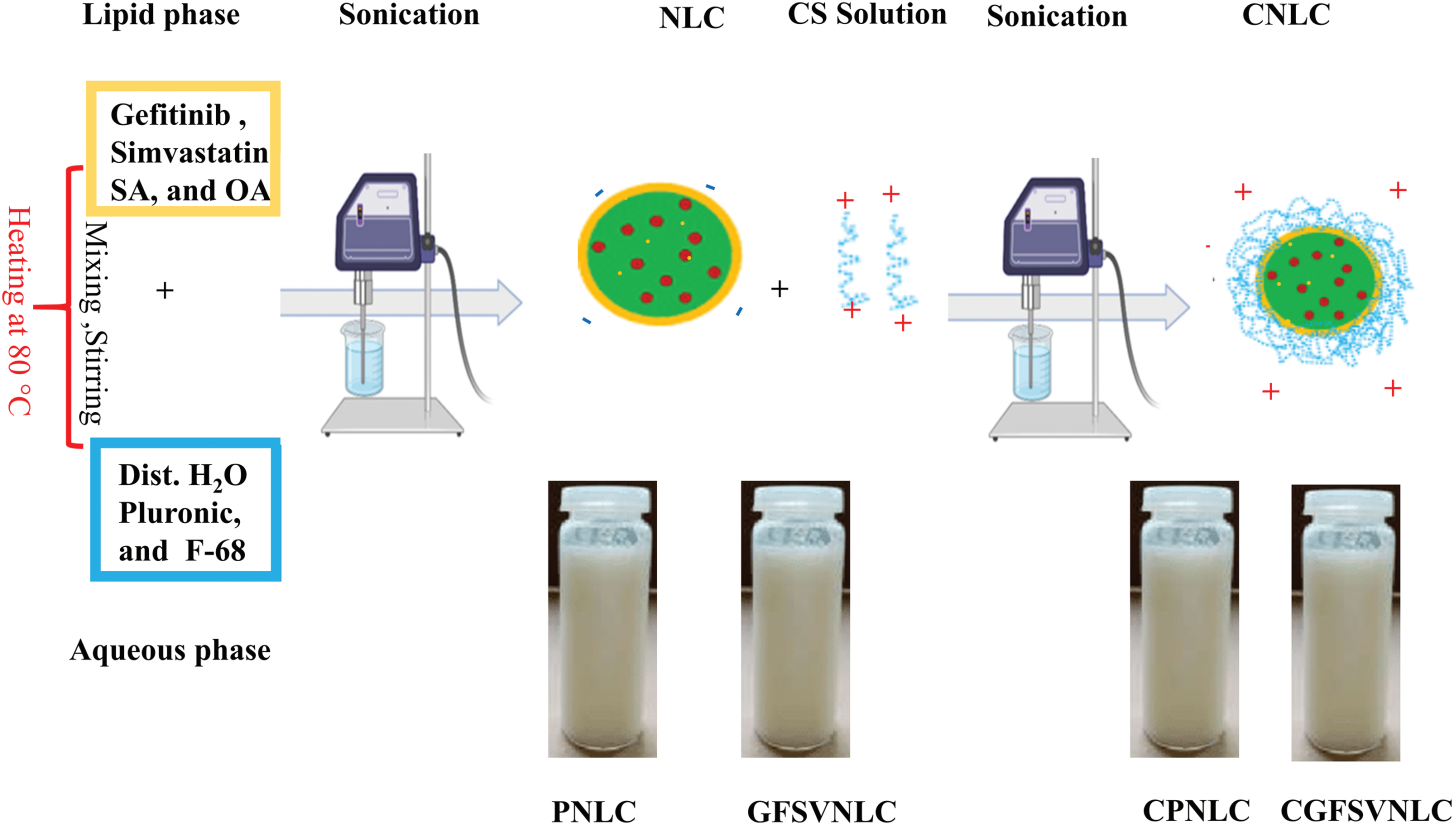
Preparation and characterization of different NLC formulations: Dawn-top, top-dawn assembly, and morphological appearance of PNLC, GFSVNLC, CPNLC, and CGFSVNLC. The primary components of the lipid and aqueous phases were small molecules of very minute size. Upon mixing of the aqueous phase and lipid phase microemulsion was formed in micro-size. Upon sonication, the NLCs were formed in nanosized.

In the present study, plain NLCs **(**PNLCs), GFSV-loaded NLCs (GFSVNLCs), CS-coated PNLCs (CPNLCs), and CS-coated GFSVNLCs (CGFSVNLCs) were assembled using ultrasonic melt-emulsification method. This is a down-top, top-down technique for gathering lipid and aqueous phases into NLCs. The construction of NLCs was indicated by the milky appearance of the preparations, see Fig. 1. In the ultrasonic melt-emulsification method, organic solvents aren’t used, and a small quantity of surfactant is present. Therefore, NLCs are expected to be devoid of deleterious effects on living cells and could be intended for biological studies. Table S1 displays the composition and the role of each ingredient of PNLCs, SVGFNLC, CPNLC, and CSVGFNLC.

### Impact of PS and PDI of NLCs

The existing results indicated that the prepared NLCs have a nanosize range with PDI in the desired range, these findings confirmed that the prepared NLC cargoes are consistent, see Table S2. In the present work the size of the NLCs is the range of (253–349 nm), therefore, GFSV NLC formulations are expected to be actively imported by the HepG2 cells. Table S2 indicates the PS and PDI of different NLC preparations.

### Impact of NLCs ZP

In the present study, the engineering of the NLC surface by CS induces cationic shift as an approach for enhancement of CGFSVNLC uptake by the HepG2 cell line. In the present results, the ZP of the different NLC preparations is in the desired range and shifted from anionic to cationic by CS capping. Moreover, PS, PDI, and ZP do not change for 2 months, this indicates the stability of NLC preparations, see Table S2.

### Biocompatibility of NLCs

**Figure 2:**
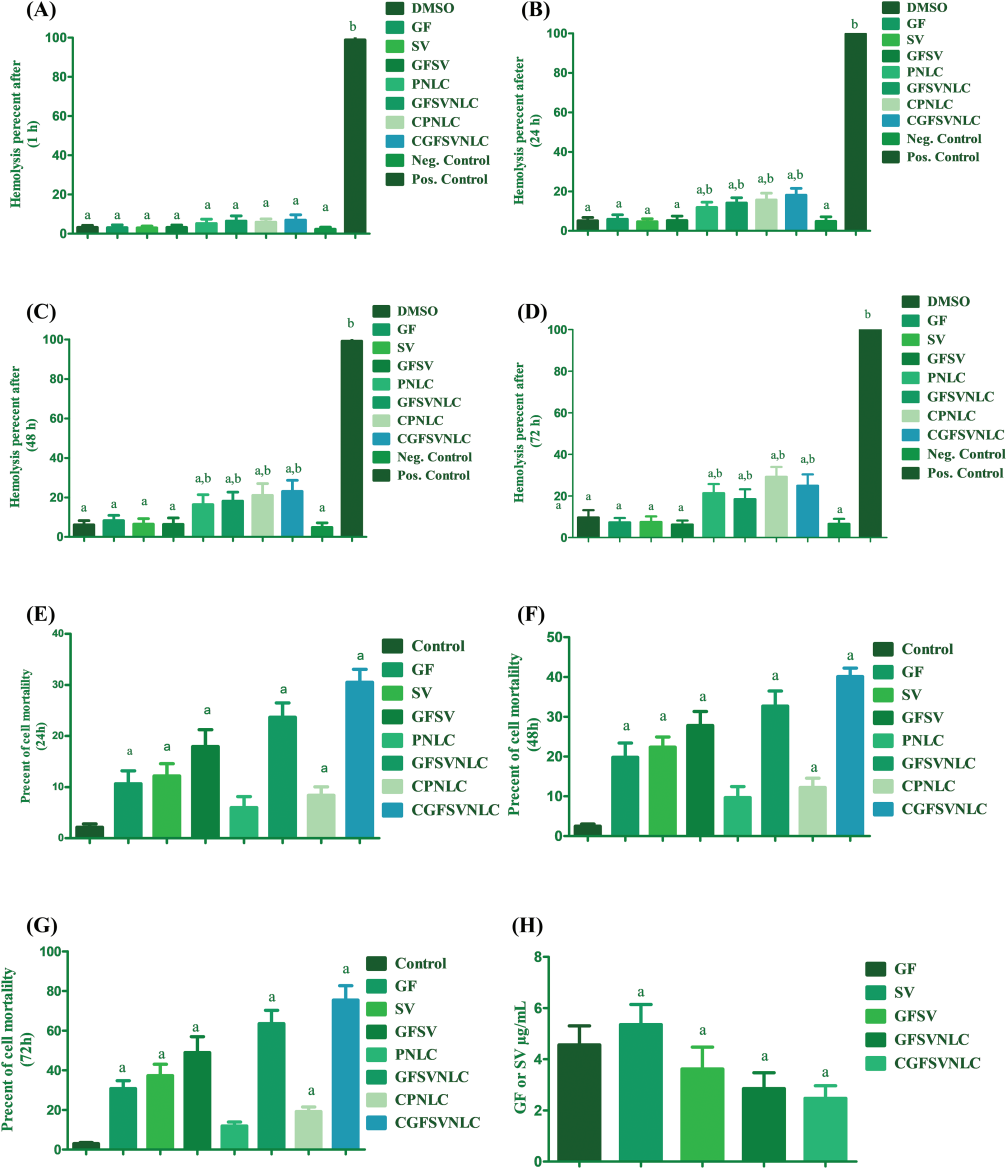
Biocompatibility and cytotoxicity of DMSO, GF, SV, GFSV, as well as PNLC, GFSVNLC, CPNLC, and CGFSVNLC formulation. (A–D) The effect of different NLC formulations on erythrocytes hemolysis after, 1, 24, 48, and 72 h incubation, respectively. Superscript letters indicated when significant differences were observed from either negative control or positive control. a: significant decrease from positive control complete hemolysis. b: significant increase from negative control (no hemolysis), *p*-value < 0.0001. (E–G) Depict the percent of HepG2 mortality upon treatment with DMSO, GF, SV, GFSV, PNLC, GFSVNLC, CPNLC, and CGFSVNLC for 24, 48, and 72 h treatment. (H) Depicts IC50 of free GF, SV, GFSV combination, and drug-loaded NLC formulations. a: significant decrease from control HepG2 untreated cells. b: significant increase from control HepG2 untreated cells. c: significant increase from control HepG2 untreated cells. *p*-value < 0.0001. One-way analysis of variance was used for data analysis; Tukey’s posttest was used to determine the statistical differences between groups. The data were expressed as mean ± SD, 6 samples per group.

In the present study, the biocompatibility of DMSO (1%), GF, SV, and GFSV in DMSO (1%), as well as PNLC, GFSVNLC, CPNLC, and GFSVNLC was investigated using erythrocytes suspension. The hemolysis percent as compared with positive and negative control was a marker for the biosafety of NLC cargoes as a critical issue. NLC ingredients include SA, OA, surfactants, and CS, which might affect red blood cell integrity. The present results indicated that the hemolysis percent of all prepared NLCs was about 5%, these results are similar to the negative control, after 1 h. On the contrary, after 24, 48, and 72 h incubation of PNLC, GFSV NLC, CPNLC, and CGFSVNLC with erythrocytes suspension, the hemolysis percent was moderately increased compared to negative control and still markedly low compared to positive control. Despite observed hemolysis at 24, 48, and 72 h, the hemolysis percent does not exceed 30 percent, Fig. 2A–D displays these results.

### Effect of NLCs on HepG2 cell mortality

The current results showed that MTT-formazan formation by HepG2 cells is declined upon treatment with GF, SV, GFSV, PNLC, GFSVNLC, CPNLC, and CGFSVNLC compared to the 1% DMSO treated group as reference for control viable cells. Interestingly, the mortality percent was augmented by the time upon treatment with SVGF combination, and GFSVNLC. Furthermore, CS capping of NLCs improves cell mortality compared with other treated groups. Fig. 2E–G demonstrates the effect of different formulations of HepG2 cell mortality during 24, 48, and 72 h incubation time. Additionally, the GFSV combination and loading of GFSV in NLCs decreases the IC50 of GF and SV (Fig. 2H).

### Effect of GFSVNLCs on HepG2 apoptosis

**Figure 3:**
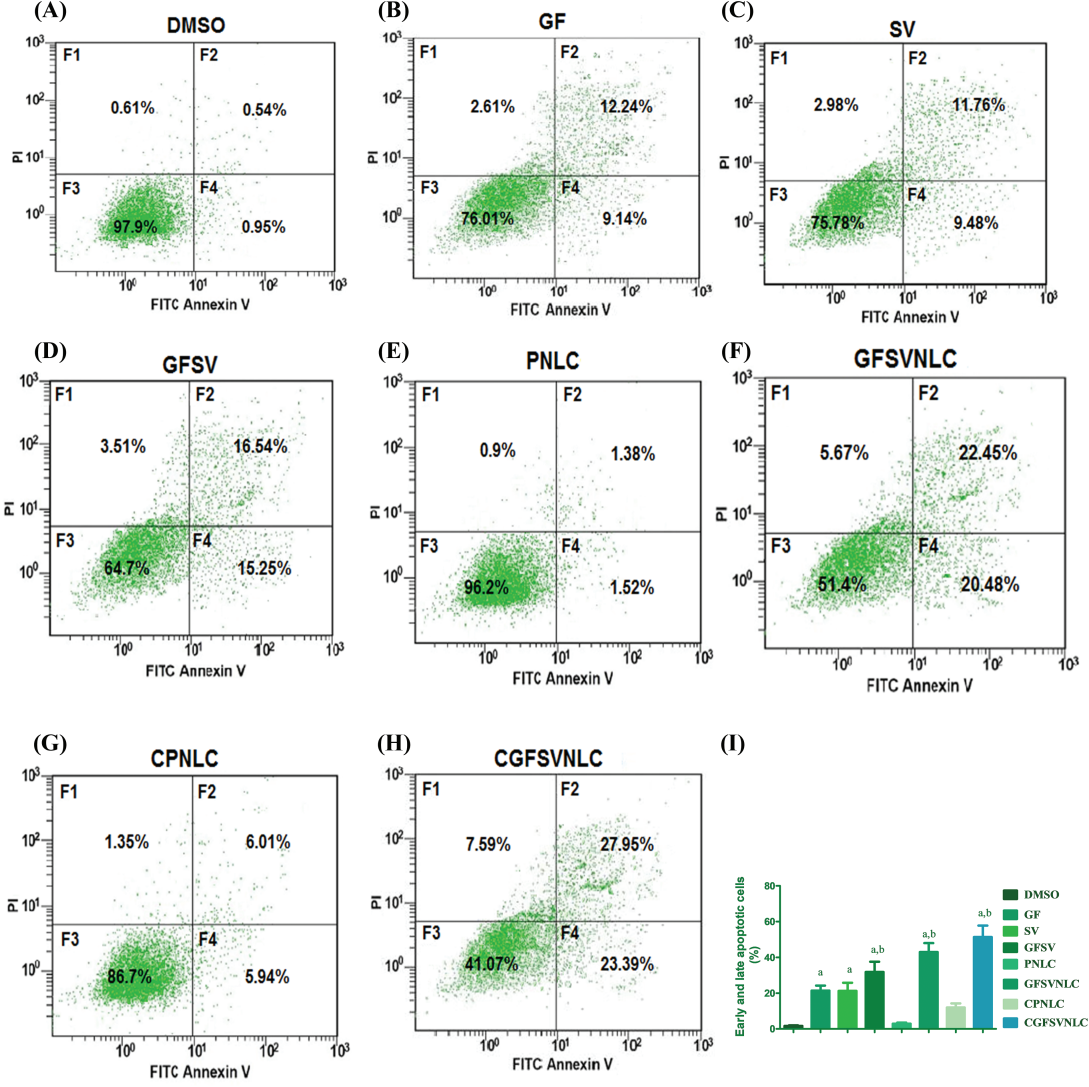
Representative flow cytometry images of Annexin V-FITC/propidium iodide double-staining and the percent of the apoptotic cell upon exposure to DMSO, GF, SV, GFSV, PNLC, GFSVNLC, CPNLC, and CGFSVNLC (A–H), respectively. The fourth quadrant represents damaged cells (F1), late apoptotic (F2), living cells (F3), and early apoptotic cells (F4). (I) Depicts the effect of DMSO, GF, SV, GFSV, PNLC, GFSVNLC, CPNLC, and CGFSVNLC on the percent of early and late apoptotic cells Superscript letters indicated when significant differences were observed from either control or treated HepG2 cells. a: significant increase from control HepG2 untreated cells. b: significant increase from GF or HepG2 treated cells, *p*-value < 0.0001. One-way analysis of variance was used for data analysis; Tukey’s posttest was used to determine the statistical differences between groups. The data were expressed as mean ± SD, 6 samples per group.

The existing study revealed that the percent of the apoptotic cells of the HepG2 cell line was increased upon exposure to pure drugs GF, SV, and GFSV combination as well as by treatment with PNLCs, GFSVNLCs, CPNLCs, and CGFSVNLCs. Interestingly, the GFSV combination, GFSVNLC, and CGFSVNLC treated groups showed a pronounced percentage of apoptotic cells compared to other groups, Fig. 3 represents flow cytometry images of Annexin V-FITC/propidium iodide double-staining and the percent of the apoptotic cell upon exposure to DMSO, GF, SV, GFSV, PNLC, GFSVNLC, CPNLC, and CGFSVNLC (Fig. 3A–H), respectively. Moreover, Fig. 3I displays the comparisons between these groups.

### Molecular Docking Simulation

#### JNK3

**Figure 4:**
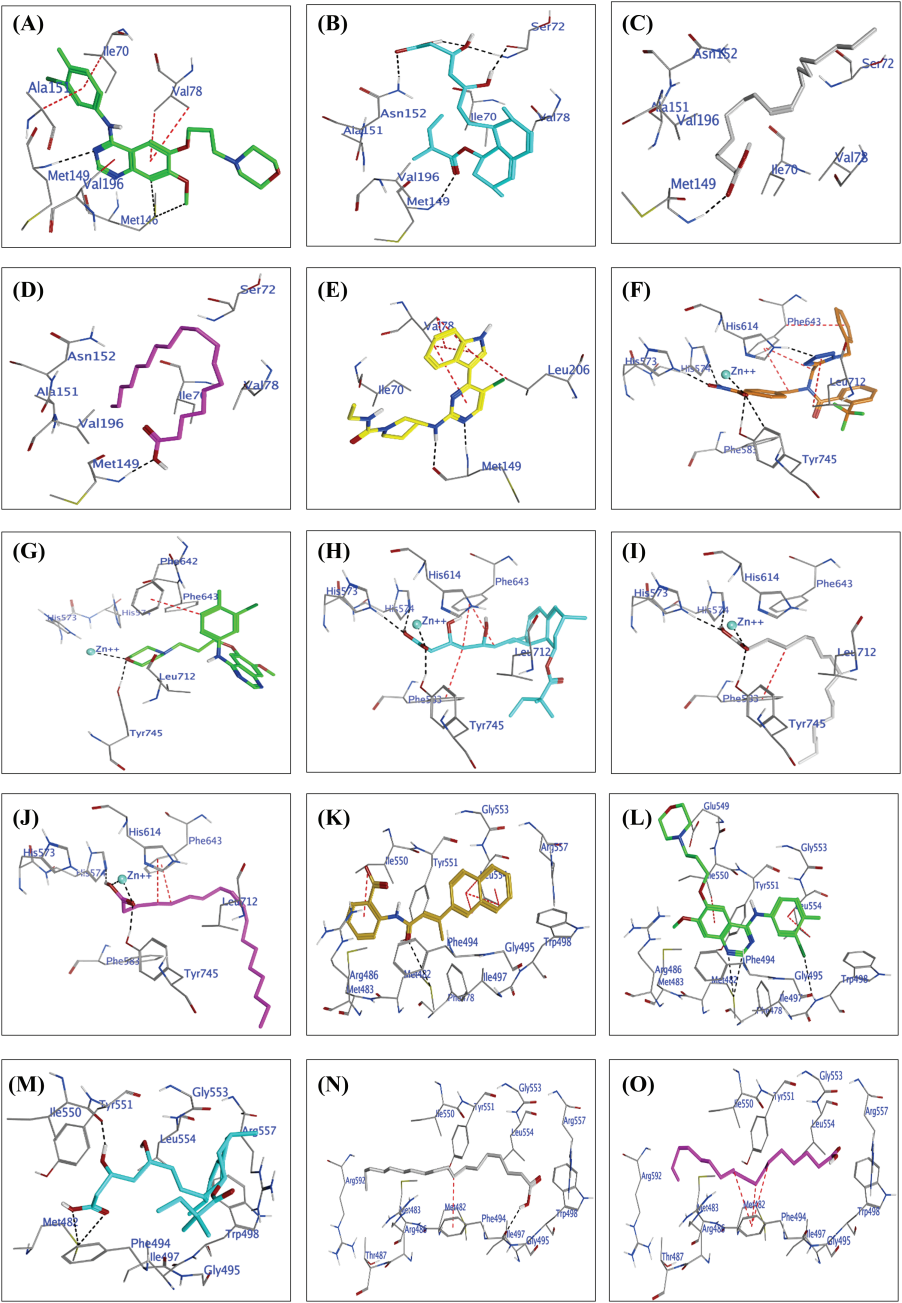
(A to E): Docked conformations of inhibitors in the JNK3 enzyme active site (PDB: 2P33). Panels (A) to (E) display Gefitinib (green), tenivastatin (cyan), Stearic acid (grey), Oleic acid (purple), and the co-crystalline ligand (2P33, yellow), respectively, highlighting key residue interactions. (F to J): Docked conformations of inhibitors in the Histone deacetylases enzyme (HDAC6) active site (PDB: 6PYE). Panels (F) to (J) display the co-crystalline ligand (6PYE, brown), Gefitinib (green), tenivastatin (cyan), Stearic acid (grey), and Oleic acid (purple), respectively, highlighting key residue interactions. (K to O): Docked conformations of inhibitors in the telomerase enzyme active site (PDB: 5CQG). Panels (K) to (O) display the co-crystalline ligand (55C, gold), Gefitinib (green), tenivastatin (cyan), Stearic acid (grey), and Oleic acid (purple), respectively, highlighting key residue interactions.

In the present study, the docking results GF, tenivastatin, SA, OA, and the co-crystalline ligand (2P33) with the JNK3 macromolecule can be reinterpreted concerning the structural details of JNK3 as follows**, **see Fig. 4A–E**, **Table S3. Concerning GF, in the present results GF exhibits multiple interactions with the N-terminal kinase domain, particularly with MET 146 and MET 149, through H-donor and H-acceptor bonds, indicative of its affinity for the ATP-binding site. The aromatic 6-ring of Gefitinib forms π-H interactions with residues in proximity to the glycine-rich nucleotide-binding sequence, like ILE 70 and VAL 78, suggesting an alignment with the phosphate anchor loop. These interactions span the ATP-binding site and the activation loop, influencing the positioning of the phosphorylation lip (Fig. 4A, Table S3). Tenivastatin is the active metabolite of SV, in the present results tenivastatin docking interactions with JNK primarily involve H-acceptor bonds with SER 72, MET 149, and ASN 152, highlighting its orientation towards the active site. Its interactions suggest that it may affect the alignment of the catalytic loop and potentially influence the positioning of the DFG loop, given its proximity to the phosphorylation lip (Fig. 4B, Table S3).

Additionally, SA and OA interact with MET 149 as an H-acceptor, implicating a significant binding within the active site, potentially affecting the nucleotide-binding due to their close distance to the adenine base binding region. The binding scores imply that these acids could perturb the ATP-binding site’s integrity, particularly interacting near the domain interface, which involves key residues like Ile70 and Val78, (Fig. 4C,D, Table S3).

Co-crystalline ligand (2P33): As expected, the co-crystalline ligand exhibits a pattern of interactions, including H-donor and H-acceptor bonds with MET 149, closely mimicking the natural ATP interactions within the JNK3 active site. The π-H interactions with VAL 78 suggest an involvement with the glycine-rich phosphate anchor loop. The co-crystalline ligand likely maintains the structural integrity of the ATP-binding site, contrasting with the other test compounds which may induce changes in the conformation of the active site (Fig. 4E, Table S3).

By interpreting the docking results within the context of JNK3’s structural regions and loops, we can hypothesize that the test compounds exhibit varying degrees of affinity and potentially different mechanisms of action based on their interactions with the ATP-binding site, the phosphorylation lip, and the surrounding loops that are critical for JNK3’s activity. These results provide a foundation for understanding how these compounds might modulate JNK3’s function and guide further structural optimization for potential JNK3 inhibitors.

#### HDAC6

The docking results for the test compounds GF, tenivastatin, SA, OA, and the reference co-crystalline ligand (6PYE) against HDAC6 show varied interactions and binding affinities within the enzyme’s active site, which is characterized by a tube-like channel leading to a catalytic Zn^2+^ ion at its base, surrounded by a dynamic internal cavity (Fig. 4F–J, Table S4). The co-crystalline ligand (6PYE) displays a strong binding affinity with a score of −12.25 kcal/mol, forming multiple ionic and metal interactions with the Zn^2+^ ion and hydrogen bonds with HIS 573, HIS 574, and TYR 745. These interactions are likely stabilized by the tubular active site structure and the dynamic cavity which facilitates the accommodation of the ligand and assists in the removal of byproducts post-deacetylation (Fig. 4F, Table S4).

The present results revealed that GF shows a moderate binding score of −6.506 kcal/mol, engaging with TYR 745 and the Zn^2+^ ion through hydrogen bonds, and forming π-interactions with PHE 642. These interactions may influence the catalytic loop and the accessibility to the active site due to their proximity to the dynamic Ser30–Lys36 loop and the conserved HIS and ASP residues that coordinate the Zn^2+^ ion (Fig. 4G, Table S4). However, tenivastatin presents a higher binding affinity of −8.041 kcal/mol compared to GF, with hydrogen bond donors and acceptors interacting with HIS 573, HIS 574, TYR 745, and metal contacts with the Zn^2+^. Its binding pattern suggests an engagement with the active site that could perturb the catalytic mechanism, potentially affecting the deacetylation process and the dynamics of the internal cavity (Fig. 4H, Table S4). Moreover, SA exhibits a binding score of −8.55 kcal/mol, forming hydrogen bonds with HIS 574 and HIS 573, and interacting with the Zn^2+^ ion. Its interactions within the active site hint at the potential to alter the dynamic cavity’s conformation, which is critical for substrate processing and product release (Fig. 4I, Table S4). Similarly, OA has a binding affinity of −7.84 kcal/mol, also utilizing hydrogen bonds with HIS 574 and TYR 745, as well as metal interactions with the Zn^2+^ ion. Similar to the other compounds, Oleic acid’s docking suggests involvement with the core structural features of the enzyme, which may affect the enzyme’s function due to its interactions with residues around the Zn^2+^ ion and the active site channel (Fig. 4J, Table S4).

These docking results, interpreted within the context of HDAC6’s structure, suggest that each compound could potentially interfere with the enzyme’s function through distinct mechanisms, influenced by their unique interactions with the catalytic Zn, the surrounding residues, and the active site’s structural dynamics.

### Telomerase

The docking results of the compounds GF, tenivastatin, SA, OA, and the co-crystalline ligand (55C) with telomerase (PDB: 5CQG) provide insights into their potential interactions and affinities with the enzyme, see Fig. 4K–O. Co-crystalline Ligand (55C) shows a strong binding score (−7.484 kcal/mol), suggesting a high affinity for the telomerase active site. This ligand forms a hydrogen bond with MET 482 and exhibits significant pi-H interactions with residues ILE 550 and LEU 554. The interactions with LEU 554, particularly through multiple residues in its 6-ring structure, indicate a strong fit within the active site that may stabilize the ligand within the telomerase structure, (Table S5 and Fig. 4K).

In the current investigation, GF demonstrates a binding score of −7.457 kcal/mol, also indicating a strong affinity. It interacts through hydrogen bonds with MET 482 and pi-H interactions with residues ILE 550 and LEU 554, which are part of the telomerase’s structure crucial for ligand binding. The pi-pi interactions observed with PHE 494 suggest an additional stabilizing interaction, which could be significant for the inhibitory function of Gefitinib on telomerase, (Table S5 and Fig. 4L). Furthermore, tenivastatin has a binding score of −7.123 kcal/mol, again showing strong interactions with MET 482 through hydrogen bonds. The hydrogen bond with ILE 550 and the binding score indicate that tenivastatin could effectively compete with the natural substrates of the enzyme (Table S5 and Fig. 4M). SA has a somewhat lower binding score of −6.921 kcal/mol compared to the previous compounds. Its hydrogen bond with PHE 494 and pi-H interaction with the same residue suggests a favorable interaction within the telomerase active site, potentially influencing the enzyme’s function (Table S5 and Fig. 4N). OA exhibits the lowest binding score among the tested compounds (−6.57 kcal/mol) but still suggests moderate binding affinity. Its interactions are mainly through pi-H contacts with PHE 494, which are likely to contribute to its positioning within the telomerase active site (Table S5 and Fig. 4O).

Overall, the compounds show interactions predominantly with MET 482, a residue that may be crucial for the catalytic activity of telomerase. The pi-H and pi-pi interactions with residues ILE 550, LEU 554, and PHE 494 suggest that these ligands could influence the binding and processing of telomerase’s natural substrate. These docking results, taken together, may provide a starting point for the design of new inhibitors targeting the telomerase active site.

## Discussion

Nanomedicine is a promising approach to overcome the drawbacks of classical methods of tumor therapy [32]. Frequently the cancer cells resist traditional therapy due to MDR through many mechanisms including drug inactivation and escape programmed cell death machinery including apoptosis, ferroptosis, pyroptosis, and oncolysis [4]. Additionally, cancer cells modify drug targets and metabolism at genetic and epigenetic levels [4]. Thus, molecular hybridization of medicines or assembly of hybrid drug delivery cargos in the term of CCT is suggested as a solution to MDR by tumor cells [3,5]. Certainly, CCT could block multiple cascades involved in the hysterical growth and spread of cancer cells [3]. Consequently, CCT is expected to enhance cancer patients’ compliance. Logically, CCT could induce a synergistic effect as a hopeful therapeutic strategy in the cancer MDR battle [3]. Thus, CCT could resolve MDR as the foremost problem in the management of HCC [1].

In this study, NLCs are harnessing to deliver GF and SV into HCC, exactly, cancer cells are greedy for lipids uptake [33]. Moreover, lipids are essential elements for cancer cells’ progression and development of tumor mass. Hence, cancer cells exhibit a high demand for lipid uptake in terms of cholesterol, FAs, and other lipids [33]. Consequently, in a cancer situation, most of the circulated blood lipids are uptake by the cancer cells [33]. NLCs are enriched with SA and OA, thus GFSVNLC cargoes are promising strategies for HCC drug targeting. In the current study, SA and OA are the main lipids components of NLCs, these FAs are the intermediate of phospholipids as a main building block for cancer cell membranes [46]. Moreover, FA are the precursors of mediators that are essential regulators of cell signaling involved in cell growth [47]. Therefore, NLCs were documented as cancer delivery cargoes [48]. Additionally, the internalization of NLCs has cancer cell tropism and active uptake by several lipid uptake receptors overexpressed by the cancer cells [33,49]. Stimulatingly, FAs have a therapeutic impact on cancer cell proliferation at genetic and epigenetic machinery [50]. Truly, CS induces cationic corona around NLCs with boosted drug trafficking into the cancer cells [40,51]. In the current study, CS was added to form a cationic cap on the NLC’s exterior. Therefore, the cellular uptake and intracellular deposition of CGFSVNLC are expected to induce drug-targeting capability [34].

Indeed, The NLCs have a propensity to be gathered in cancer cells by the EPR effect and active machinery [28,32,50]. NLCs selectively target the tumor cells by the EPR effect due to nanoscale size, likewise, the ingredients of NLCs promote active delivery into the cancer cells [52]. Therefore, GFSV NLCs could be selectively trapped in HCC by EPR. However, free GF and SV diffuse passively into the intracellular milieu of normal and cancer cells in none non-selective manner [34]. Classically, pure GF and SV are small lipophilic drugs, therefore, they are fellow passive transport machinery without difference between cancer and normal cells. In the present study, GF and SV are loaded into NLCs, thus the import of both drugs into the cell is shifted to large active transport. Herein, GFSVNLC could be selectively picked up by HCC by active machinery. Accordingly, GFSVNLC cargoes are promising for selective uptake by cancer cells. Likewise, NLCs are suggested to enhance their delivery of GF and SV into cancer cells [5,28]. Thus, NLCs are nanometers in size and are internalized by the cells via receptor-mediated endocytosis [34].

From the drug delivery point of view, ZP influences the cellular uptake of NLCs [34]. Interestingly, cationic NLCs favorably interact with the plasma membranes with ionic interaction [53]. This is attributed to the membranes of living cells having a negative charge due to the presence of sialic acid and negatively charged lipids. Thus, cationic NLCs could induce high cellular import compared with neutral and anionic ones with high therapeutic impact [34]. Likewise, ample studies reported that cationic NLCs induce marked cell death compared to neutral and anionic cargoes [34]. This is accredited to cationic NLCs increasing the fluidity of cell membranes and enhancing cellular uptake [34]. Moreover, anionic NLCs are rapidly cleared from systemic circulation [54]. Therefore, the engineering of the NLC surface is vigorous to withstand the drug for a longer time in the blood as an approach to improving the therapeutic effectiveness [54].

As well, ZP can improve the targeting and the liberation of drugs at specific sites in a selective manner [54]. Additionally, ZP dictates the machinery of cellular trafficking of NLCs cellular uptake mechanisms, accordingly, cationic NLCs are internalized into the intracellular environment via macropinocytosis machinery. On the contrary, anionic NLCs are imported intracellularly by clathrin-/caveolae-independent endocytosis machinery [34]. This attribute, CS contains amino groups that form cationic charges around negatively charged lipids as a major component of NLCs [40]. Likewise, ample studies demonstrated that the cationic NLCs induced active drug delivery [53]. Furthermore, another study indicated that the cationic cargoes increase the accumulation of SV in HepG2 cell lines [27]. This is attributed to the cationic charge of CS interaction with the cell membrane by the electrostatic interaction, van Dear Waal force, and non-ionic interaction [55].

Additionally, ZP has a role in NLC stability, however, the stability of nanocarriers depends on the balance between two counteracting forces including van der Waals, and the electrical double layer [53]. The differential light scattering technique was selected for the measurement of ZP of NDCs [53]. The NLCs that have neutral or slightly charged ZP values tend to form aggregate [53]. Such cargo is rapidly recognized and massively removed upon administration *in vivo* [53]. Hence, high ZP values the vital to ensure stability and avoid aggregation as well as for the extension of plasma half-live of NLCs [53]. Importantly, ZP can be controlled by stabilizer concentration, or by surface engineering by anionic or cationic agents [53].

The erythrocytes are an appropriate model for biocompatibility studies of NLCs due to their abundance, sensitivity, durability, and lack of intracellular contents [56]. The erythrotoxicity in terms of changes in erythrocytes’ appearance, viability, contents, hemolysis, or behavior can give evidence about the biocompatibility of NLCs [56]. Principally, NLCs that produced 5% hemolysis of erythrocytes are tolerable for administration *in vivo* [57]. Consequently, erythrocyte hemolysis is a critical parameter in evaluating the biosafety of NLCs intended for biomedical applications [58]. Taken together, the erythrocyte hemolysis rate confirmed the biocompatibility of the pharmaceutical formulations containing polymers, surfactants lipids, or other additive materials [59].

These results are in agreement with ample studies that demonstrated the gentle effect of lipid nanomaterials on erythrocyte hemolysis [34,57]. As well, another study confirmed the gentle effect of lipid nanoparticles on erythrocytes [60]. Moreover, it has been reported that lipid-based nanocarriers are safe for *in vivo* applications [61].

The observed hemolysis by increasing the incubation time (24, 48, and 72 h) might be attributed to the electrostatic interactions of CNLC with erythrocytes membrane. Consequently, hemolysis erythrocytes occurred by increasing the incubation time [58]. Similarly, it has been documented that cationic NLCs elicit more interaction with biomembranes compared to anionic carriers [34]. Therefore, in the present investigations, the hemolysis percent of CS-engineered NLCs is greater than CS-free NLCs with or without drug loading.

The slight hemolysis percent indicates the precise amount of ingredients of surfactants, CS, and lipid phase in PNLC, GFSVNLC, CPNLCs, and CGFSVNLC. The current observations are synchronized with published work demonstrating that lipid nanoparticles are hemocompatible and safe in biological systems [33,40]. Taken together, in the present investigations, the gentle effect of NLCs on erythrocytes indicates their biosafety with little erythrotoxicity over a long period. Therefore, the prepared NLCs could be intended for biological applications. Accordingly, PNLC, GFSVNLC, CPNLC, and CGFSVNLC are appropriate for cytotoxicity studies on the HepG2 cell line.

In the present study, the MTT assay was used as an indicator of cell mortality using the HepG2 cell line as a surrogate model for HCC. MTT assay is a spectrophotometric method that investigates the mitochondrial function as an essential organelle for cell survival [62]. The current results confirmed that the GFSV combination induced a synergistic effect on HepG2 cell mortality compared to control, free GF, and SV. Moreover, the increase in cell death percent by the time indicated the sustained release of GF and SV from NLCs and CS-coated NLCs. The existing findings agree with several studies established that the codelivery of GF and SV induced a synergistic effect on cancer cell death [1,5,8,28,55]. Likewise, the combination of statins and TKIs as CCT enhanced cancer cell mortality with increased drug sensitivity and inhibited MDR [63]. Thus, statins induced synergistic effects with other anticancer agents including TKIs [11,64].

The synergistic effect between GF and SV might be attributed to a similar effect on kinases and modulating the cell’s proliferation signal cascades. However, ample studies reported that the effects of statins correspond to the effects of TKI members on malignant cells [12]. Interestingly, the cytotoxic effect of GF and SV chimera is enhanced upon loading into NLCs as well as by CS-coated NLCs. These results are concurrent with other investigations indicating that drug-loaded NLCs improve the therapeutic impact of anticancer agents [65]. Likewise, abundant studies demonstrated that CS nanocarriers, CS-coated drug carriers, and GFSV codelivery enhance the cytotoxicity of GF and SV [1,5,27,66]. The enhancement of HepG2 mortality by CGFSVNLC indicates the therapeutic impact of NLC ingredients. In this regard, cationic NLCs are anticipated to enhance cellular uptake with marked HCC death [34].

Indeed, cationic NLCs elicit electrostatic interaction and liquify the cell membranes [34]. Thus, CS-capped lipid drug cargoes induced a manifest increase in HepG2 cell death [40]. Moreover, CS chelates cholesterol as an essential element for the membrane assembly of highly proliferative cancer cells [40]. However, CS creates a cationic corona on NDCs that augments cellular uptake of medications into HepG2 cells [40,66]. Similarly, another study established that CS-decorated SV-loaded nanoparticles provoked HepG2 killing [27]. The electrostatic attraction of the positively charged ammonium group of CS and the negatively charged phosphate of phospholipids or sialic acid on the HepG2 cell membrane mediate the cellular uptake and cytotoxicity of GFSV [55].

Apoptosis, ferroptosis, necroptosis, and pyroptosis are different forms of cell death associated with inflammatory responses for termination of the cell life [67]. These forms of cell death contribute to oncogenesis and malignant transformation [67]. Hence, targeting apoptosis, ferroptosis, necroptosis, and pyroptosis regulatory mechanisms is an innovative insight for the management of cancers [67]. Specifically, these programmed cell death machinery are controlled by many genes that have a central part in the growth, homeostasis, and pathophysiology [67]. It has been documented that NDCs inhibit the growth of HepG2 cells by triggering apoptosis, necrosis, ferroptosis, and oncolysis [67]. In this context, there are several studies indicating that SV modulates ample genes that affect apoptosis [68]. Another study attributed the cytotoxic effect of statins to apoptosis, ferroptosis, and pyroptosis [25]. Moreover, a previous study demonstrated that SV induces apoptosis by cholesterol diminution and mitochondrial malfunction. Furthermore, SV causes HepG2 cell cycle arrest by induction of apoptotic proteins [12,27]. As well, SV elicits negative impacts on ubiquinone biosynthesis, ATP production, and posttranslational modification of oncogenes [69], thus SV inhibits cell proliferation through the blocking of multiple cascades involved in cell division [69]. Furthermore, several reports indicated that statins could trigger programmed cell death in tumor cell lines [69].

It has been reported that SV induces cell death by targeting the JNK signaling pathway. These conclusions confirmed SV has a potential role in the inhibition of MDR by cancer cells [70]. In the present study, the MDS suggested that GF, SV, SA, and OA modulate the activity of JNK3, HDAC6, and telomerase. In this context, GF, SV and NLC components (SA, OA, and CS) enhance HepG2 cell death by modulation of apoptosis. These effects are attributed to inhibition of JNK3, HDAC6, and telomerase activities through direct binding with the active sites or chelation of Mg and Zn ions as essential cofactors for these enzymes. Thus, the codelivery of GF and SV in NLCs is suggested to increase drug sensitivity, decrease drug dosing, and overcome MDR. Consequently, the CCT approach could induce the synergistic effect by targeting multiple signaling pathways involved in HepG2 cell survival. These findings are concurrent with several studies reported that inhibitors of telomerase activity as a mechanism for cancer cell killing. Similarly, other studies indicated that TKIs have inhibitory effects on telomerase activity [71]. In the same way, statins were documented as telomerase inhibitors by interfering with the telomere/telomerase machinery [72]. Specifically, statin-induced inhibition of telomerase activity in HepG2 cells in cell lines [73]. Additionally, FAs were reported as inhibitors of telomerase [74]. Likewise, FAs trigger cell death through lipotoxicity, apoptosis, and necrosis [75]. Moreover, FAs induce HDAC inhibition, mitochondrial malfunction, and peroxisome proliferation, as well as activate caspases and mediate beta-defensin production [50].

**Figure 5:**
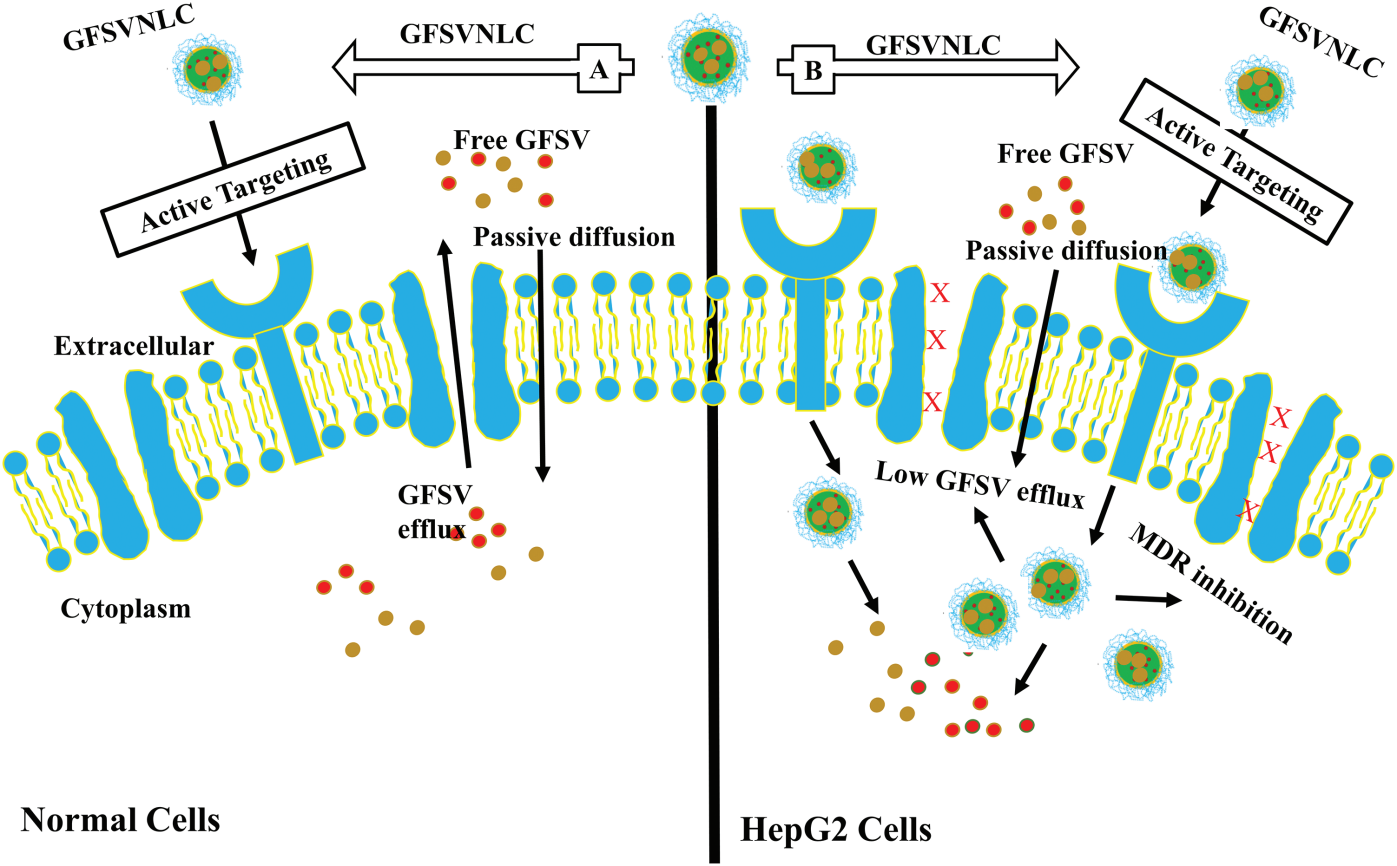
NLCs mediate active targeting of GF and SV into HepG2 cells. A: Normal Cells: GFSVNLC are less introduced into normal cells, however, LDLR overexpression is low compared to HCC. Thus, GF and SV are delivered into normal cells at low levels with minimal side effects. B: HCC: GFSVNLC cargoes are highly introduced into HCC due to LDLR overexpression. Thus, GF and SV are highly and selectively delivered into HCC at high concentrations with extraordinary therapeutic effects.

HCC cells overexpress LDL-r, thus, CGFSVNLC could be actively delivered into HepG2 cells by a receptor-mediated endocytosis mechanism [76]. GFSVNLCs are highly introduced into HCC due to LDLR overexpression. Thus, GF and SV are highly and selectively delivered into HCC at high concentrations with extraordinary therapeutic effects, see Fig. 5. Moreover, positive ZP improves the internalization of GF and SV into HepG2 cells [34]. This attribute, CS capping induces cationic charges around negatively charged lipids as a major component of NLCs [40]. Likewise, ample studies demonstrated that the cationic NLCs induced active drug delivery [53]. This increased the accumulation of GF and SV in HepG2 cell lines [27].

Additionally, van Dear Waal force and non-ionic interaction are involved in the cellular uptake of NDCs [55]. Collectively, both GF and SV are highly accumulated into HepG2 cells. SV is suggested as chemo-preventive, monotherapy for cancer, or in combination TKIs augmented HCC mortality [76]. Consequently, the codelivery of GF and SV in CS-capped NLCs could induce a synergistic effect to increase the sensitivity of cancer cells to chemotherapy and overcome MDR [77]. This assumption is supported by ample literature reporting the synergistic effect of statins and TKIs and their role in the allowance of HCC patient’s survival [78]. The cytotoxic effect of statins could be attributed to inhibition of cholesterol production, growth signal inhibition, mitochondrial malfunction, ATP depletion, free radical production, and apoptosis [79]. Specifically, statins block G-protein biomembranes anchoring with inhibiting Ras signaling [80]. Prominently, lipid raft is a key player in statin-mediated inhibition of tumor growth and migration. Specifically, SV reduced tumor cell growth, cellular cholesterol levels, cholesterol content in lipid rafts, and membrane integrity [81].

MDR is a major challenge in cancer control, it is characterized by tumor relapse and metastasis [81]. Indeed, SV can reverse cholesterol-induced MDR through the modulation of lipid rafts caveolae and ABC transporters [82]. Ample studies indicated that statins and TKIs co-delivery produce a synergistic overcoming MDR in ample tumors [69]. Therefore, the combination of SV and anticancer medicines improves patients’ survival with decreased mortality rates [82]. SV re-sensitized lung cancer cells to paclitaxel resistance. Moreover, GF and SV codelivery enhanced apoptosis in GF-resistant lung cancer cells [81].

Finally, the current work indicated that GFSV combination alone or GFSV-loaded NLCs are suggested to reduce tumor cell proliferation, and migration and trigger the apoptotic signal. In this regard, numerous clinical trials of SV and TKIs stated that a combination could improve the cancer patient’s life [10]. Codelivery of SV and GF increases cancer cell sensitivity, lowers drug dosing, and lessens MDR. The synergistic effect of GF and SV may be attributed to the similarity in the mechanism of action on kinases, telomerase, and HDACs [69]. Consequently, the GFSV combination was exploited to trigger further cell death pathways with definite cytotoxic effects on tumor cells [69]. Additionally, both drugs are substrates to the same metabolizing enzymes, this could explain the synergistic effect of GF and SV combination [70]. These findings documented the CCT opens a new avenue to expand the therapeutic impact of SV and TKI members in HCC management [63].

The major limitations of this study, are the cytotoxic and apoptotic effects inspected using the HepG2 cell line as a model for HCC. Therefore, testing of GFSV-loaded NLCs on patient-derived samples could provide a more comprehensive understanding of their efficacy. Additionally, this study lacks *in vivo* experiments, thus investigating the effect of the CGFSVNLCs in animal models of HCC is critical to assess their therapeutic potential impact and translate the findings to clinical application.

Additionally, this study investigated only three molecular targets (JNK3, HDAC6, and telomerase). Investigative the impact on a broader range of signaling pathways and cellular processes involved in HCC pathogenesis and drug resistance at genetic and epigenetic levels would provide a better understanding of the mechanism of action for GF and SV. The study did not compare the efficacy of the CGFSVNLCs to other clinically used therapies for HCC, which would be necessary to assess the potential clinical relevance and advantages of the proposed approach. These limitations open avenues for future research plans about CCT and nanomedicines for the treatment of MDR hepatic cancer.

## Conclusion

The study findings concluded that the prepared NLCs are consistently nano-sized. The addition of CS shifts the ZP of GFSVNLC from anionic to cationic in CGFSVNLC. PNLC, GFSVNLC, CPNLC, and CGFSVNLC are all shown to be biocompatible. Treatment with SVGF combination, GFSVNLC, and CGFSVNLC significantly increases HepG2 cell mortality. Particularly, CGFSVNLC demonstrates a pronounced effect with reduced IC50 compared to other groups. Additionally, exposure to pure drugs and their combinations with GFSV increases the percentage of apoptotic HepG2 cells, with notable effects seen in the GFSV combination, GFSVNLC, and CGFSVNLC groups. Molecular docking studies suggest that GF, SV, SA, and OA modulate JNK3, HDAC6, and telomerase activities by interacting with active sites and cofactors. These results shed light on the anticancer mechanisms of GF and SV, along with the ingredients of NLCs, which inhibit JNK3, HDAC6, and telomerase—enzymes often over-activated in cancer cells. Consequently, CGFSVNLC appears promising for reducing GF and SV dosages while increasing cancer cell drug sensitivity and MDR.

## Supplementary Materials











## Availability of Date and Materials

All data are embedded in the manuscript.

## Supplementary Materials

The supplementary material is available online at https://doi.org/10.32604/or.2024.053337.
