# Supplementary material for: Chitosan capped-NLCs enhanced codelivery of gefitinib and simvastatin into MDR HCC: impact of compositions on cell death, JNK3, and Telomerase
Source: Oncol Res. 2025 Jan 16;33(2):477–92. doi: 10.32604/or.2024.053337 (PMC11754001; doi:10.32604/or.2024.053337)
Supplement: Supplementary file 1 [file OncolRes-33-53337-s001.docx]

**Table S1:** The composition of PNLC, GFSVNLC, CPNLC and CGFSVNLC.

| Ingredients | Role | PNLC | GFSVNLC | CPNLC | CGFSVNLC |
| --- | --- | --- | --- | --- | --- |
| Stearic acid (mg) | Solid lipid | 900 | 900 | 900 | 900 |
| Oleic acid (mg) | Liquid lipid | 200 | 200 | 200 | 200 |
| Simvastatin (μg) | API | - | 50 | - | 50 |
| Gefitinib (μg) | API | - | 50 | - | 50 |
| Pluronic F-68 (mg) | Surfactant | 500 | 500 | 500 | 500 |
| Distilled water (mL) | Solvent | 48 mL | 48 mL | 48 mL | 48 mL |
| CS solution (mL) | Cationic Coat | - | - | Equal volume | Equal volume |
