# Supplementary material for: Chitosan capped-NLCs enhanced codelivery of gefitinib and simvastatin into MDR HCC: impact of compositions on cell death, JNK3, and Telomerase
Source: Oncol Res. 2025 Jan 16;33(2):477–92. doi: 10.32604/or.2024.053337 (PMC11754001; doi:10.32604/or.2024.053337)
Supplement: Supplementary file 2 [file OncolRes-33-53337-s002.docx]

**Table S2:** The particle size, zeta potential, polydispersity index, and drug content of PNLC, GFSVNLC, CPNLC, and CGFSVNLC.

|  | PNLC | GFSVNLC | CPNLC | CGFSVNLC |
| --- | --- | --- | --- | --- |
| Particle size (nm) | 243.0 ± 52.00 | 267.0 ± 63.00 | 312.0 ± 73.0 | 339.0 ± 68.0 |
| Zeta potential (mV) | -27.00 ± (-3.00) | -29.0 ± (-2.00) | 21.00 ± 2.00 | 23 .0 ± 3.00 |
| Polydispersity index | 0.200 ± 0.034 | 0.311 ± 0.040 | 0.240 ± 0.020 | 0.307 ± 0.100 |
| GFSV content (mg/mL) | - | 50 + 50 | - | 50 + 50 |

Data were expressed as the mean ± SD, N=3.
