# Supplementary material for: Chitosan capped-NLCs enhanced codelivery of gefitinib and simvastatin into MDR HCC: impact of compositions on cell death, JNK3, and Telomerase
Source: Oncol Res. 2025 Jan 16;33(2):477–92. doi: 10.32604/or.2024.053337 (PMC11754001; doi:10.32604/or.2024.053337)
Supplement: Supplementary file 3 [file OncolRes-33-53337-s003.docx]

**Table S3:** Docking analysis of Gefitinib, Tenivastatin, Stearic acid, Oleic acid, and co-crystalline ligand (2P33) with JNK3 (PDB: 2P33), showing interaction types, distances, energies, and binding scores to predict binding affinities within the enzyme's active site.

| **Compounds** | **Ligand** | **Receptor** | **Interaction** | **Distance** | **E (kcal/mol)** | **Binding score (kcal/mol)** |
| --- | --- | --- | --- | --- | --- | --- |
| Gefitinib | C 28 | SD MET 146 (A) | H-donor | 3.96 | -0.3 | -7.683 |
|  | C 32 | SD MET 146 (A) | H-donor | 4.12 | -0.5 |  |
|  | N 38 | N MET 149 (A) | H-acceptor | 3.61 | -0.8 |  |
|  | 6-ring | CB ILE 70 (A) | pi-H | 4.14 | -0.5 |  |
|  | 6-ring | CG1 VAL 78 (A) | pi-H | 4.25 | -0.7 |  |
|  | 6-ring | CG2 VAL 78 (A) | pi-H | 4.67 | -0.3 |  |
|  | 6-ring | CA ALA 151 (A) | pi-H | 4.14 | -0.8 |  |
|  | 6-ring | CG1 VAL 196 (A) | pi-H | 4.83 | -0.3 |  |
| Tenivastatin | O 66 | O SER 72 (A) | H-donor | 3.07 | -0.5 | -5.982 |
|  | O 18 | N MET 149 (A) | H-acceptor | 3.57 | -0.6 |  |
|  | O 61 | ND2 ASN 152 (A) | H-acceptor | 3.41 | -0.7 |  |
|  | O 64 | N SER 72 (A) | H-acceptor | 3 | -2.7 |  |
| Stearic acid | O 55 | N MET 149 (A) | H-acceptor | 2.97 | -1.1 | -6.222 |
| Oleic acid | O 54 | N MET 149 (A) | H-acceptor | 3.17 | -1.9 | -6.521 |
| Co-crystalline ligand (2P33) | N12 27 | O MET 149 (A) | H-donor | 3.22 | -1 | -7.255 |
|  | N27 48 | O HOH 603 (A) | H-donor | 2.75 | -2 |  |
|  | N18 35 | N MET 149 (A) | H-acceptor | 3.18 | -4.1 |  |
|  | 5-ring | CG1 VAL 78 (A) | pi-H | 4.39 | -0.4 |  |
|  | 6-ring | CG1 VAL 78 (A) | pi-H | 4.82 | -0.3 |  |
|  | 5-ring | CG2 VAL 78 (A) | pi-H | 3.9 | -1 |  |
|  | 6-ring | CG2 VAL 78 (A) | pi-H | 4.14 | -0.4 |  |
|  | 5-ring | CD1 LEU 206 (A) | pi-H | 3.74 | -0.7 |  |
