# Supplementary material for: Chitosan capped-NLCs enhanced codelivery of gefitinib and simvastatin into MDR HCC: impact of compositions on cell death, JNK3, and Telomerase
Source: Oncol Res. 2025 Jan 16;33(2):477–92. doi: 10.32604/or.2024.053337 (PMC11754001; doi:10.32604/or.2024.053337)
Supplement: Supplementary file 4 [file OncolRes-33-53337-s004.docx]

**Table S4:** Comparative docking interactions and affinities of Gefitinib, Tenivastatin, Stearic acid, Oleic acid, and the co-crystalline ligand (6PYE) with the HDAC6 enzyme (PDB: 6PYE). The ligand-receptor interactions, including hydrogen bonding, metal coordination, and π-interactions, with corresponding distances and energies, culminate in the overall binding scores for each compound.

| **Compounds** | **Ligand** | **Receptor** | **Interaction** | **Distance** | **E (kcal/mol)** | **Binding score (kcal/mol)** |
| --- | --- | --- | --- | --- | --- | --- |
| Co-crystalline ligand (6PYE) | N36 53 | NE2 HIS 574 (A) | H-donor | 2.92 | -0.7 | -12.25 |
|  | N12 2 | NE2 HIS 614 (A) | H-acceptor | 3.35 | -2 |  |
|  | O01 55 | CE2 TYR 745 (A) | H-acceptor | 3.08 | -0.4 |  |
|  | O01 55 | OH TYR 745 (A) | H-acceptor | 2.78 | -0.8 |  |
|  | O37 57 | NE2 HIS 573 (A) | H-acceptor | 2.85 | -6 |  |
|  | O01 55 | ZN ZN 804 (A) | Metal | 2.26 | -3.1 |  |
|  | O01 55 | ZN ZN 804 (A) | Ionic | 2.26 | -11.9 |  |
|  | O37 57 | NE2 HIS 573 (A) | Ionic | 2.85 | -5.5 |  |
|  | O37 57 | ZN ZN 804 (A) | Ionic | 1.96 | -17 |  |
|  | C07 21 | 6-ring PHE 643 (A) | H-pi | 4.05 | -0.8 |  |
|  | C09 24 | 6-ring PHE 643 (A) | H-pi | 4.26 | -0.3 |  |
|  | 6-ring | CB PHE 643 (A) | pi-H | 4.63 | -0.4 |  |
|  | 5-ring | CD1 LEU 712 (A) | pi-H | 4.48 | -0.6 |  |
|  | 6-ring | 6-ring PHE 583 (A) | pi-pi | 3.79 | 0 |  |
| Gefitinib | O 7 | OH TYR 745 (A) | H-acceptor | 3.08 | -1.3 | -6.506 |
|  | O 7 | ZN ZN 804 (A) | H-acceptor | 2.84 | -0.4 |  |
|  | C 52 | 6-ring PHE 642 (A) | H-pi | 4.28 | -0.5 |  |
| Tenivastatin | O 62 | NE2 HIS 574 (A) | H-donor | 2.78 | -1.4 | -8.041 |
|  | O 61 | OH TYR 745 (A) | H-acceptor | 2.71 | -1.1 |  |
|  | O 62 | NE2 HIS 573 (A) | H-acceptor | 3.06 | -1.6 |  |
|  | O 61 | ZN ZN 804 (A) | Metal | 2.03 | -2.7 |  |
|  | C 47 | 6-ring PHE 643 (A) | H-pi | 3.94 | -0.5 |  |
|  | C 52 | 6-ring PHE 583 (A) | H-pi | 4 | -0.4 |  |
|  | C 52 | 6-ring PHE 643 (A) | H-pi | 4.03 | -0.3 |  |
| Stearic acid | O 55 | NE2 HIS 574 (A) | H-donor | 2.77 | -2.6 | -8.55 |
|  | O 54 | OH TYR 745 (A) | H-acceptor | 2.75 | -1.1 |  |
|  | O 55 | NE2 HIS 573 (A) | H-acceptor | 3.16 | -0.9 |  |
|  | O 54 | ZN ZN 804 (A) | Metal | 2.17 | -2.8 |  |
|  | C 44 | 6-ring PHE 583 (A) | H-pi | 4.55 | -0.3 |  |
| Oleic acid | O 52 | NE2 HIS 574 (A) | H-donor | 2.76 | -2.1 | -7.84 |
|  | O 52 | ZN ZN 804 (A) | H-acceptor | 2.26 | -0.6 |  |
|  | O 54 | OH TYR 745 (A) | H-acceptor | 2.77 | -1.6 |  |
|  | O 54 | ZN ZN 804 (A) | Metal | 2.11 | -3.2 |  |
|  | C 39 | 6-ring PHE 643 (A) | H-pi | 3.87 | -0.3 |  |
|  | C 42 | 5-ring HIS 614 (A) | H-pi | 4.03 | -1 |  |
