# Supplementary material for: Chitosan capped-NLCs enhanced codelivery of gefitinib and simvastatin into MDR HCC: impact of compositions on cell death, JNK3, and Telomerase
Source: Oncol Res. 2025 Jan 16;33(2):477–92. doi: 10.32604/or.2024.053337 (PMC11754001; doi:10.32604/or.2024.053337)
Supplement: Supplementary file 5 [file OncolRes-33-53337-s005.docx]

**Table S5:** Comparative docking interactions and affinities of test compounds Gefitinib, tenivastatin, Stearic acid, Oleic acid, and the co-crystalline ligand (55C) with the telomerase enzyme (PDB: 5CQG). The table details ligand-receptor interactions, including hydrogen bonding, metal coordination, and π-interactions, with corresponding distances and energies, culminating in the overall binding scores for each compound.

| Compound | Ligand | Receptor | Interaction | Distance | E (kcal/mol) | Binding score (kcal/mol) |
| --- | --- | --- | --- | --- | --- | --- |
| Co-crystalline ligand (55C) | O21 10 | SD MET 482 (A) | H-donor | 3.95 | -0.5 | -7.48432922 |
|  | O23 24 | O HOH 784 (A) | H-acceptor | 2.81 | -1.7 |  |
|  | 6-ring | CG1 ILE 550 (A) | pi-H | 4.29 | -0.3 |  |
|  | 6-ring | CB LEU 554 (A) | pi-H | 3.88 | -0.6 |  |
|  | 6-ring | CD1 LEU 554 (A) | pi-H | 4.61 | -0.3 |  |
|  | 6-ring | CD1 LEU 554 (A) | pi-H | 4.08 | -0.7 |  |
|  | 6-ring | CD2 LEU 554 (A) | pi-H | 4.34 | -0.7 |  |
| Gefitinib | N 35 | SD MET 482 (A) | H-donor | 3.58 | -0.9 | -7.457 |
|  | C 36 | SD MET 482 (A) | H-donor | 3.54 | -0.3 |  |
|  | CL 49 | O GLY 495 (A) | H-donor | 3.5 | -0.4 |  |
|  | 6-ring | CG2 ILE 550 (A) | pi-H | 3.67 | -0.3 |  |
|  | 6-ring | CB LEU 554 (A) | pi-H | 4.03 | -0.6 |  |
|  | 6-ring | CD1 LEU 554 (A) | pi-H | 4.44 | -0.5 |  |
|  | 6-ring | 6-ring PHE 494 (A) | pi-pi | 3.7 | 0 |  |
|  | 6-ring | 6-ring PHE 494 (A) | pi-pi | 3.93 | 0 |  |
| Tenivastatin | O 61 | SD MET 482 (A) | H-donor | 3.74 | -0.8 | -7.123 |
|  | O 62 | SD MET 482 (A) | H-donor | 3.77 | -0.4 |  |
|  | O 64 | O ILE 550 (A) | H-donor | 2.65 | -1.1 |  |
| Stearic acid | O 55 | O PHE 494 (A) | H-donor | 3.06 | -1.3 | -6.921 |
|  | C 32 | 6-ring PHE 494 (A) | H-pi | 3.69 | -0.3 |  |
| Oleic acid | C 23 | 6-ring PHE 494 (A) | H-pi | 4.1 | -0.3 | -6.57 |
|  | C 30 | 6-ring PHE 494 (A) | H-pi | 3.55 | -0.4 |  |
|  | C 36 | 6-ring PHE 494 (A) | H-pi | 4.54 | -0.3 |  |
